# Epicardial Ablation For Ventricular Tachycardia

**DOI:** 10.1016/s0972-6292(16)30564-2

**Published:** 2012-12-02

**Authors:** Giuseppe Maccabelli, Hiroya Mizuno, Paolo Della Bella

**Affiliations:** 1 Arrhythmia Department and Clinical Electrophysiology Laboratories, Ospedale San Raffaele - IRCCS- Milan - Italy; 2 Department of Advanced Cardiovascular Therapeutics, Osaka University Graduate School of Medicine, Osaka Japan

**Keywords:** epicardial ablation, ventricular tachycardia

## Abstract

Epicardial ablation has lately become a necessary tool to approach some ventricular tachycardias in different types of cardiomyopathy. Its diffusion is now limited to a few high volume centers not because of the difficulty of the pericardial puncture but since it requires high competence not only in the VT ablation field but also in knowing and recognizing the possible complications each of which require a careful treatment.

This article will review the state of the art of epicardial ablation with special attention to the procedural aspects and to the possible selection criteria of the patients

## Introduction

Epicardial ablation, introduced in 1996 for a very selected patient population, has become over the years, an intriguing technique for the treatment of ventricular tachycardia(VT) in different cardiomyopathies. Currently used only in few high volume centers with the subxyphoidal puncture, the epicardial ablation is performed mainly after a previously failed endocardial approach and only in few cases as a first choice.

The very limited experience available for this procedure probably explains why it is included in the last Expert Consensus on Catheter Ablation of Ventricular Arrhythmias [1] as a possible way of treatment of VT in different settings without, however, including it in the statements of the guidelines.

This article will review the "state of the art" of epicardial ablation with special attention to the procedural aspects and to the possible selection criteria of the patients.

## Technical considerations

### How to perform pericardial access

The subxyphoidal pericardial puncture is currently performed using the technique originally proposed by Sosa et al [2].

**Figure 1:**
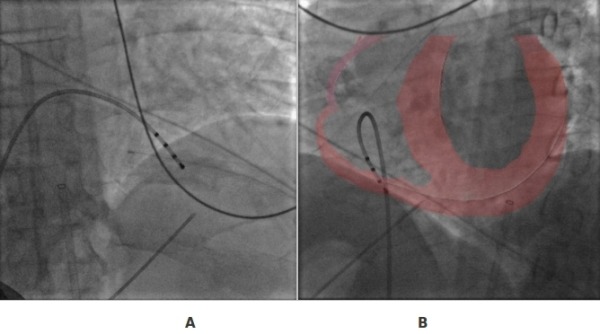
A: direction of the needle in RAO view B: direction of the needle in LAO view. See text for explanations

Starting from the border of the subxyphoidal process, a Tuohy needle is advanced toward the left shoulder at 45º angle from the skin's surface and at the same angle from the middle line of the sternum. To a more precise evaluation of the anatomy and of the position of the heart inside the thorax, one catheter in right ventricle and one in the coronary sinus may be useful references. To this point a continuous check in RAO and LAO view (Figure 1) with the X-ray is recommended during the advancement of the needle tip toward the cardiac silhouette.Starting from the border of the subxyphoidal process, a Tuohy needle is advanced toward the left shoulder at 45º angle from the skin's surface and at the same angle from the middle line of the sternum. To a more precise evaluation of the anatomy and of the position of the heart inside the thorax, one catheter in right ventricle and one in the coronary sinus may be useful references. To this point a continuous check in RAO and LAO view (Figure 1) with the X-ray is recommended during the advancement of the needle tip toward the cardiac silhouette.

**Figure 2:**
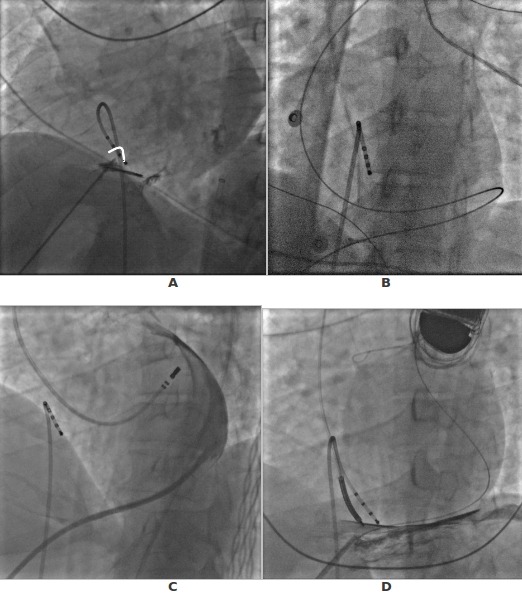
a: pericardial tenting during epicardial puncture. B: the presence of the wire that goes from left to right is the safest way to be sure that the wire is in the pericardial space and not in a ventricular chamber c: injection of the contrast before remove the wire is important to be sure that the distal part of the sheath is in the pericardial space and not outside like in d. The presence of the wire allows the reinserction of another sheath in cases like D where the sheath is outside the pericardial space

To understand at best the position of the needle, some operators use a small amount of contrast media to image "pericardial tenting" like in the transeptal puncture (Figure 2). Once the needle is in the pericardial space, conventional Seldinger technique is used to introduce a 9F sheath that allows not only the insertion of the mapping catheter in the pericardial space but also, through the side port, the drainage of fluids introduced with the irrigated catheter. For this approach we prefer to use a long sheath (23 cm.) instead of the conventional one of 11 cm, because using the latter increases the possibility to lose the access, particularly in patients with a large thorax. To reduce this risk it is strong recommended, before removing the wire, to verify the position of the sheath with the injection in the pericardial space of a small amount of contrast media (Figure 2).

In some cases the selective approach of the anterior wall of the right ventricle may be useful instead of the usual inferior-left lateral wall of the heart. This can be obtained using a smaller angle than the conventional 45º angle to the skin surface. To overcome this need in the last few months we have used a steerable sheath specially designed for the pericardial approach (Agilis Epi@ St. Jude Medical) which permits the easy achievement of all areas warranting a stable contact with the epicardial surface.

We usually remove the sheath at the end of the procedure if epicardial ablation was not performed and if there is no blood in the drainage system. Otherwise, to avoid unintentional damage of the myocardial wall, a pigtail is left in place. In this case the sheath is removed, after an echo check to exclude the presence of pericardial fluid 24 hours later.

It is important to remember, before removing the sheath, the injection of corticosteroids (triamcinolone acetonide 2 mg/kg) to prevent post-procedure pericarditis.

Any pericardial procedure must be carefully planned and performed always before the endocardial approach and therefore before starting with the anticoagulation therapy.

There are some cases where the subxypoidal puncture or the epicardial mapping is not possible due to the presence of inflammatory pericardial adherence. In this situation we usually call the surgeon who carries out a Marfan window which simply permits to reach the epicardial surface and the removal of the adherence when present. The Marfan window is performed with a 4-5 cm cutaneous incision just below the xyphoid process; adhesions are carefully divided, an 8 French sheath is advanced in the pericardial space and finally the parietal layer of the pericardium is closed.

In infrequent cases (concomitant heart surgery, inefficacy of RF energy - epicardial fat -, impossibility to perform RF ablation - adherence, presence of coronary arteries or phrenic nerve -, impossibility to perform the pericardial puncture) the ablation can be performed with open chest in the operating theatre using the CARTO system and the cryo-energy. In this setting, due to the difficulty to induce the clinical arrhythmia and for the impossibility to compare the ECG morphology, a sinus rhythm based mapping and ablation procedure will be applied.

## Complications

Evaluating complications occurring during an epicardial ablation we have to differentiate between those which can occur during the subxyphoidal puncture, during the ablation procedure and after the procedure.

Among the possible complications that can occur during the puncture, we have to bear in mind that damage of all subdiafragmatic organs can occur. Damage of the liver, stomach or colon may occur particularly in prone subjects. Patients with right heart decompensation or with megacolon, for example, have to be carefully evaluated before the procedure if necessary also with a CT scan. Two cases of abdominal bleeding due to damage of a diaphragmatic vessel are reported in the literature [3]. One [4] of these occurred in our center two years ago and was discovered 3 days after the procedure because of the presence of progressive anemia in the absence of pericardial effusion and without any other significant abdominal symptoms. In both cases abdominal surgery was required to control the bleeding.

Another possible complication occurring during the puncture is the pleural catheterization with guidewire reported in one study population in 1.5% of cases that usually occurs without pneumothorax or complications.

Main cause of epicardial bleeding may be due to damage to the myocardial wall (usually the right ventricle) or to a coronary artery occurring during the puncture or during RF ablation. This complication is reported in 30% of cases in the study population of Sosa and coll [3] and usually doesn't preclude the procedure. In more recent publications [3,4], a lower incidence is reported (between 3.7 and 4.5). The simple puncture of the right ventricle is not usually followed by pericardial blood effusion. This complication called "dry right ventricle puncture" is reported in 4.5% to 17% of cases and will definitely decrease as skill improves. To avoid more important damage to the myocardial wall during the puncture it is really important, in doubtful cases, to check the position of the tip of the needle with a small amount of contrast before inserting the guidewire and in any case to insert it very gently.

A common minor complication is the postprocedure pericarditis with different degrees of severity that usually cause postprocedure chest pain (21% of cases [4]). To reduce the occurrence of this complication it is important to remove the pericardial sheath as soon as possible and the routine pericardial infusion of steroids before removing it.

### Coronary arteries

Another important problem that we can encounter during epicardial ablation is due to the presence of the epicardial vessels. Although larger vessels are probably protected by the blood flow [6] it was empirically suggested to consider 12 mm as the minimum optimal distance of the ablating tip catheter from the coronary vessel. Nevertheless average distance reported in the study of Della Bella [4] is 8.6±7.8 mm and in the last guidelines is strictly suggested to deliver RF energy maintaining a minimum distance > 5 mm from the coronary artery [1].

It is always stronglyrecommended to perform a coronary angiography to clarify the position of the tip of the ablating catheter compared to the vessel. For the difficulty to visualize in a 3D way an object of which we have only two-dimensional images, the concurrent use of a 3D electro-anatomical map may be a useful tool with which we can annotate over the map tags displaying the course and position of the coronary artery.

**Figure 3:**
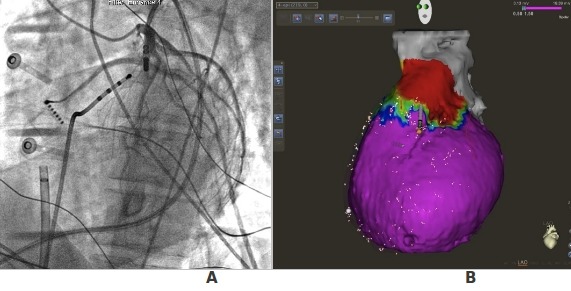
A: Coronary angiography performed with the ablating catheter in a site where, during VT, a diastolic potential was registered. The tip of the catheter is close to the LAD artery. B: Carto bipolar map merged with the epicardial anatomy obtained with a CT. Catheter tip (arrow) is shown in the same position of that registered during angiography.

Another helpful possibility is provided by the use of merge capabilities of the mapping system that allows the superimposition over the electro-anatomical map of the anatomical images obtained with a CT (Figure 3). In frequent cases, with the surface rendering tools included in the system, a clear visualization of the major coronary arteries can be obtained.

In the most important published experiences damage to a coronary artery is reported in one case by Sosa [3] and in one by Sacher [5]. No damage was reported in Della Bella's survey [4]. Although all recommend the use of coronary angiography it is interesting to note that in the last paper it was only performed in 43% of cases, with a wide variation among centers (15% - 87%). Maybe this is due to the fact that in some cases, particularly in the lateral wall of the right ventricle or in big true scar areas, there are regions where the likelihood to find major coronary arteries is very low and if the operators feel sure of themselves the coronary angiography can be avoided.

In addition, the use of anatomical images obtained with CT scan and the merge process available with the CARTO technology could explain the reduced recourse to the angiography.

### Phrenic nerve

The position and the course of the phrenic nerve may in some cases be an important obstacle to the delivery of RF energy. While the damage to the right phrenic nerve that usually descends along the superior vena cava and along the right atrium is described occurring during ablations of atrial arrhythmias [7,8], the left may be damaged during the ablation of VT. This nerve usually runs along the lateral wall of the left ventricle but many different positions have been described [9]. Different strategies have been proposed over the years to localize the course of the nerve and to avoid damage during RF delivery in site near the nerve. The latter are usually described as a case report or in small groups of patients but none has been systematically evaluated in big populations. Even if the course of the phrenic nerve could be identified with imaging technique, the only easily feasible way to detect the presence of the nerve is the use of high intensity pacing that must be performed checking the presence of the diaphragmatic capture also with X-Ray [10]. Care must be taken to perform this test, in case of intubated patients, after antagonization of any muscle relaxants.

## Specific problems

### Fat

One of the most important problems in the setting of epicardial ablation is the presence of fat that can significantly reduce the efficacy of the RF energy.

Fat, normally present on the epicardial surface, is increased in patients with CAD and is correlated positively with the staging of the cardiomyopathy [11]. Moreover, in patients with CAD, fat is thicker than in patients without cardiomyopathy (4 vs 1.5 mm) [12].

Although the use of irrigation during RF application energy with a low electrode-tissue interface temperature avoids an excessive impedance rise and permits larger and deeper lesions than dry RF, fat significantly reduces its effectiveness. Using an in vitro model, Hong et al. [13] demonstrated that epicardial fat can significantly limit the efficacy of lesions induced with energy that utilizes conductive heating. D'Avila et al. [14] evaluated the difference of the lesions obtained with standard and cooled tip RF energy. Lesions created in normal epicardial tissue with standard and cooled-tip RF ablation were 3.7 mm and 6.7 mm in depth, respectively.

In areas covered by epicardial fat (3.1±1.2 mm thick), standard RF energy did not generate any significant lesions, but cooled-tip RF lesions were 4.1±2 mm in depth. In the presence of a fat layer > 3.5 mm, cooled-tip RF was unable to produce epicardial lesions.

At the moment the only way that we have to assess epicardial distribution is the use of imaging techniques like CT scan and MRI. Abbara et al.[15] studied the epicardial fat distribution and thickness in 59 patients who underwent cardiac CT for coronary artery assessment. Fat was significantly present around the AV groove and over the right ventricle. On the contrary fat thickness was small over the lateral and inferior wall of the left ventricle. Epicardial fat was also 22% greater in older patients than in young ones.

Knowing the epicardial fat distribution is important not only to determine the possibility to obtain effective lesions but also because epicardial fat may mimic scar area. Desjardins and coll. [16] in a small patient population have recently found that a fat thickness ≥ 2.8 mm resulted in voltage attenuation and could identify, with good sensitivity and specificity, areas with low bipolar voltages (<1.5 mV). Nevertheless, in an older paper D'Avila [17] found that in the presence of fat thickness <5 mm there was no significant difference in the peak-to-peak bipolar voltage amplitude, in the bipolar electrogram duration and in the pacing threshold. Fat layers > 5 mm made ventricular capture impossible even at 10 mA. Based on these data and on our experience we think that fat is probably able to mimic scar but only when thickness is really high as usually happens, in normal patients, along the AV groove.

## How to consider a patient for an epicardial ablation

### Type of cardiomyopathy

First published experiences about epicardial ablation [18-23] include, in small patient populations, different types of structural heart diseases. In those days epicardial ablation was mainly performed as a rescue procedure and almost always only after a previously failed ablation. Over the years the experience in epicardial access has been more and more increasing and, particularly in high volume centers it has become a routine procedure. The current problem, now, is not only in which type of cardiomyopathy we have to perform the epicardial ablation but, above all, when an endo-epicardial ablation must be performed as first choice. 

One cardiomyopathy in which the expression of the pathological processes may have in some cases a predominant expression not only adjacent to the valve annulus, deep in the endocardium but also in the epicardial surface is the idiopathic dilated cardiomyopathy. This condition already exposed in the past by Perlman et al. [24] has become increasingly evident over the years. In one series of IDCM patients using a combined endo-epicardial approach, Soejima et al.[22] reported a success rate of 54% during a mean follow-up of almost one year. In this study the average epicardial scar area was 37.5 ± 10.4 cm2 (range 22 to 47.3 cm2). In the five patients with both epicardial and endocardial mapping, the scar area was larger on the epicardial surface than on the endocardial surface. In another recent paper published by Cano e coll [25] twenty-two patients with IDCM underwent endo-epicardial bipolar voltage mapping and VT ablation. In the 18 patients the mean scar area was greater in the epicardium than in the endocardium (55.3 ± 33.5 cm2 vs. 22.9 ± 32.4 cm2, p < 0.01). Epicardial low-voltage areas showed 49.7% of wide split, and/or late electrograms usually not present in the reference patients (2.3%). During follow-up of 18 ± 7 months, no recurrence of VT was identified in 14 out of 18 patients (78%) with epicardial VT. These results may explain the low success rate of IDCM patients ablated only in the endocardium. In a patient population presented by Schmidt et al [26] with previously failed endocardial ablation for VT, 15 patients with IDCM underwent epicardial mapping. In this group of patients epicardial pathological potentials were found in 80%.

Another structural heart disease in which the epicardial evaluation may be capital to increase the success rate of the ablation procedure is the arrhythmogenic right ventricular dysplasia (ARVD). In order to understand the reason why epicardial access must be considered in this type of cardiomyopathy, we have to remember that this peculiar myocardial disease, characterized by replacement of myocytes by fibro-fatty tissue usually involving in the early stage the right ventricle, occurs from the epicardium to the endocardium [27]. VTs in the setting of ARVD were historically approached, in the first published papers [28-37] in the endocardium. Although a comparison of the results obtained in these studies is obviously not possible because the difference in the ablation strategy, procedural endpoints, AAD therapy and follow-up we can observe that the reported acute success is widely spread (50-90%) and the recurrence rate increases significantly over the years. Wichter et al.[33] in his patient population reported an acute success in 22 patients (73%). However, during a follow-up period of 52 ± 37 months, 18 patients (60%) suffered from VT recurrence or syncope. The event-free survival was 63% after 1, 43% after 3, and 32% after 5 years respectively. This trend, also present in other papers, can only be in a measure explained with the disease progression of the involvement of the right ventricle and, in rare cases, of the left ventricle. Interestingly enough, in none of these experiences the epicardial surface was approached. The need of this approach has been clearly initially proved in some papers in which, however, it was always considered only as a complementary approach after one or more previous failed endocardial ablations [38-40]. In one of these papers [38], 13 patients, in which endocardial ablation failed to control clinical VTs, underwent endo-epicardial mapping and ablation procedure. Regardless of the good results obtained in the follow-up (no recurrences in 77% of patients after 18±13 months) some important findings that underline the relevance of the epicardial approach in this patient population have to be underlined. In all patients the scar area was more extensive on the epicardium (95±47 versus 38±32 cm2; P<0.001) and was uniformly marked by multicomponent and late potentials. Epicardial VTs were targeted opposite normal endocardium in 10 patients (77%) and/or opposite ineffective endocardial ablation sites in 11 patients (85%). Interestingly enough the right basal wall evaluated with the electro-anatomical maps resulted thicker in ARVD patients ( > 10 mm) as compared to the reference patients without structural heart disease (5-10 mm) and to this end we have to remember that irrigated RF energy delivery in the endocardial surface is unable to produce transmural lesions. Another paper that strengthens the importance of an endo-epicardial approach in this type of cardiomyopathy has been published by Bai et al [41]. Forty-nine patients with ARVD divided into two groups underwent endocardial ablation alone (group 1) or endo-epicardial ablation (group 2). After a follow-up of at least 3 years, 52.2% of patients in group 1 and 84.6% in group 2 (p<0.029), were free from VAs or ICD therapy. Antiarrhythmic therapy was present in 78.3% in group 1 and in only 30.8% in the endo-epicardial ablation group (P<0.001). Therefore epicardial approach seems to be unavoidable for ARVD patients but we have to remember that just in this structural heart disease it is not free of problems. As pointed out by Abbara et al15 epicardial fat resulted significantly present not only around the AV groove but also around the right ventricle. It's presence could make it hard to achieve an effective result with the use of irrigated RF energy.

Curiously enough the first published studies about epicardial ablation involved predominantly patients with CAD in which, compared with the previous, the need of this approach is probably less important. The reason of this is probably due to the history of VT catheter ablation which started just in this type of patients suffering from tolerated monomophic VT. 

In the setting of a necrotic scar, the substrate for a VT is determined by the presence of surviving ventricular muscle fibers included in dense connective tissue. Myocardial fibers on the endocardial surface of infarcts or aneurysm may survive because they receive blood directly from the ventricular cavity or from the retrograde perfusion through the sinusoidal channels [42,43]. The fibers entrapped in the fibrotic tissue, have ultrastructural and consequent electrophysiological abnormalities which may form the basis for a reentry mechanism [44]. This peculiar anatomical condition may also develop in areas adjacent to the myocardial infarction where the reduced myocardial blood flow may cause subendocardial ischemia with focal necrosis and subsequent fibrosis. This condition may explain the success, obtained with the endocardial excision [45] of the surgical treatment of VT. Therefore, in this situation, it is reasonable to infer that the optimal treatment for VTs is the endocardial ablation. 

As proved in an old animal study [46], irreversible myocardial ischemic damage occurs from the subendocardium toward the subepicardium. This progression is in function of time: transmural necrosis was 38 ± 4% after 40 min, 57 ± 7% after 3 hours, 71 ± 7% after 6 hours and 85 ± 2% after 24 hours of ischemic injury. The subepicardial myocardium in relation to the infarct size has the highest likelihood of no necrosis therefore also in this condition we can suppose that it can be substrate for VT.

The recent changes in the treatment of myocardial infarction with the early reperfusion technique has modified the main characteristics of the necrotic scars with more patchy, nontransmural distribution of necrosis [47]. This condition has increased the complexity of the arrhythmic substrate increasing the number of slow conduction areas and VT exit points which can be located not only in the subendocardial scar or in the border zone of the myocardial infarction but also deep in the myocardial wall or in the subepicardial layers.

In two previous multicenter randomized trials [48,49] of endocardial VT ablation after myocardial infarction, despite of the acute results nearly half of the whole population had recurrences at the end of the follow-up.

The complexity of the substrate and the not always encouraging results of the endocardial ablation has motivated some EP laboratories to approach ischemic patients with an endo-epicardial procedure, particularly after a previously failed endocardial approach. Considering the two most important published series, in the paper of Sacher [5] 16% of the total population of ischemic patients was submitted to an endo-epicardial ablation; 90% of this group had previously been submitted to an endocardial ablation. This percentage is more reduced in the paper of Della Bella [4] where of 85 patients with CAD, 32.9% were submitted to an endo-epicardial ablation as first choice. Interestingly enough in 12.9% there was only an epicardial approach. In other series epicardial ablation was performed only after failed endocardial ablation. Schmidt et al [50] evaluated the role of epicardial ablation in different structural heart diseases after a failed endocardial approach. 11 patients had an ischemic cardiomyopathy and five an inferior scar. In another single centre experience [51] of 17 patients with CAD, 7 underwent an endo-epicardial ablation (5 with surgical approach). All three patients with a putative epicardial isthmus site had an inferior scar. In another recent paper [52] 280 patients with CAD were divided into two groups: patients without previous ablation and patients with one or more previously failed endocardial ablations underwent an endocardial ablation in the first case and an endo or endo-epicaridal ablation in the second. VT recurrence rate was surprisingly higher in the second group in which septal or inferior scars were more common.

Based on this data, although someone [53] has supposed that the presence of an inferior scar can predict a failure of an endocardial ablation and the need for an epicardial approach, we believe that the possibility to have an epicardial scar in ischemic patients, even if infrequent, is always possible independently of the previous treatment (early reperfusion or not) and as most ischemic VTs can be successfully targeted from the endocardial surface, an epicardial approach must always be considered in the presence of previously failed endocardial ablations.

### ECG criteria

Several papers have been published in the last few years suggesting different flow-charts to recognize an epicardial origin of a VT.

The first significant paper published by Berruezo e coll [54], suggested 4 different criteria with which an epicardial origin of a VT could be identified with good sensitivity and specificity. After the evaluation of the QRS morphology of endo-epicardial pacing and of 49 VTs in 9 patients (6 of whom with CAD), a pseudo deltawave ≥ 34 msec, an intrinsecoid deflection time ≥ 85 msec, an R/S complex duration ≥ 121 msec have proven to be optimal parameters to guide the recognition of an epicardial source.

A following paper [55], suggested different region-specific morphologic criteria based on the initial vector of the QRS complex. Furthermore Authors found that the criteria proposed by Berruezo are in fact useful but sensitivity and specificity are significantly different depending on the different LV regions because not only the type of the structural heart disease but also the different regions of the LV may significantly affect the initial conduction delay of the epicardial VT on which these criteria are based.

In non ischemic cardiomyopathy Valles [56] published an algorithm for identifying an epicardial origin from basal superior and lateral left ventricle VT. With the absence of a q wave in the inferior leads, a pseudo delta wave > 75 ms a maximum deflection index > 0.59 and the presence of a q wave in lead D1 are indicative of an epicardial origin.

Recently Martinek e coll [57] evaluated the morphological criteria of 63 endo/epicardial VT in patients with CAD. None of the previously proposed criteria for non ischemic patients was reliably able to identify an epicardial origin of a VT.

Considering ECG criteria we have to keep in mind that in some cases the critical part of the reentry circuits may be located in the endocardium but the broad exit is in the epicardium and on the contrary in other an endocardial VT may be maintained by an epicardial reentry circuit.

In conclusion we think that ECG criteria are certainly useful to identify an epicardial origin of a VT but they have to be used only as a last resource and always after the evaluation of the type of the cardiomyopathy and imaging.

To plan a procedure, general rules that are almost always useful are: consider the presence of: 

-1. bundle branch block: RBBB means a free wall exit site and LBBB a septal exit site. 

-2. QRS axis: inferior axis indicates a superior exit and superior axis an inferior exit site. 

- 3. the horizontal plane that can differentiate between basal and apical exit sites.

### Imaging

The use of imaging technique has progressively increased over the years. By providing detailed anatomical and metabolic information about normal and pathological myocardium, it can improve the VT ablation procedure and the results. Of the three main imaging modalities, contrast-enhanced (CE) CT and delayed-enhanced cardiac magnetic resonance imaging (DE-MRI) are able to provide very high resolution 3D images of the anatomy of the ventricles and of the myocardial scars. 3 Fluorodeoxyglucose (FDG) PET can add important metabolic information.

The first use of imaging integration (registration with the heart of 3D images obtained with CT or MRI) was first reported in animals in 2003 [58]. Ever since, with the development of CartoMerge system by Biosense Webster and the NavX Fusion by St. Jude Medical, imaging integration has been increasingly used especially in atrial fibrillation ablation where its capability to show detailed images of the left atrial anatomy has permitted more precise and effective procedures. For the same purpose imaging integration can be used to define anatomical structure of the ventricles and above all of the epicardial surface where it can show the course of the main epicardial arteries. However the most important use of imaging technique in VT ablation is connected to the capability to identify and display functional and pathological characteristics of the myocardial tissue, knowledge of which is crucial in these type of procedures. Reentry or focal VT in the setting of structural heart disease are always supported by myocardial scars. To this end the use of imaging techniques able to identify and localize in a 3D way the presence of these scars may help to plan the procedure and to reduce the procedural time.

Dickfeld et al. were the first to report this use in 2008 [59]. In this study performed in fourteen patients who underwent a PET/CT before VT ablation, Authors proved its capability to asses the left ventricle scars and border zones and the possibility to obtain supplementary scar characterization, with the integration of the 3D scars in the electro-anatomical maps.

Contrast-enhanced cardiac magnetic resonance (CE-CMR) may be currently considered as the gold standard for the evaluation of the myocardial perfusion, of the myocardial viability and of the ventricular function. Several papers [60-63] have in the past highlighted its role in ischemic patients with the analysis of the infarct size and the periferal zone to strongly predict not only the inducibility of ventricular arrhythmias but also the cardiovascular mortality. Next to this applications MRI has been increasingly used to guide ablation procedures. All published papers, which considered a small number of CAD patients, have confirmed that scar characterization obtained with high resolution MRI looks similar to that obtained with electro-anatomical mapping. Unlike the bipolar or unipolar voltage maps, MRI provides additional information about the scar geometry providing the possibility to predict the location of VT circuits. In all cases all this information can be integrated and registered with the electro-anatomical map to guide the procedure. Although images have been obtained with different algorithm and different protocol have been used for the post-processing, a common finding is that there is a constant mismatch between scar areas identified with EAM and those identified with CE-MRI. Codreanu et al. [64] found a clear mismatch of 20% in 33% of infarct areas: in 3 cases scars identified with EAM were not confirmed on MRI, in 1 EAM underestimates the scar revealed with MRI. Desjardins [65] using a cutoff value of 1.5 mV to define scar tissue found a mismatch of 29%. The observed variance between EAM and scars at MRI was justified with the poor contact of the mapping catheter with the endocardial surface, with the low density of the electroanatomical map or with the low spatial resolution of the CE-MRI technique applied. A reduction of this discrepancy has been obtained in a recent published paper [66] using a a high-density EAM and a high spatial resolution CE-CMR technique. Applying a cutoff value of 60% of the maximum pixel signal intensity to discriminate between core and border zone of the scar a very good match has been obtained between core identified with EAM and those with CE-MRI. Despite this improvement core scars identified with EAM remain slightly smaller. This may be explained, according to the Authors, by the effect of the far-field signals generated by the surrounding healthy tissue that could reduce the area where low voltages are registered.

**Figure 4:**
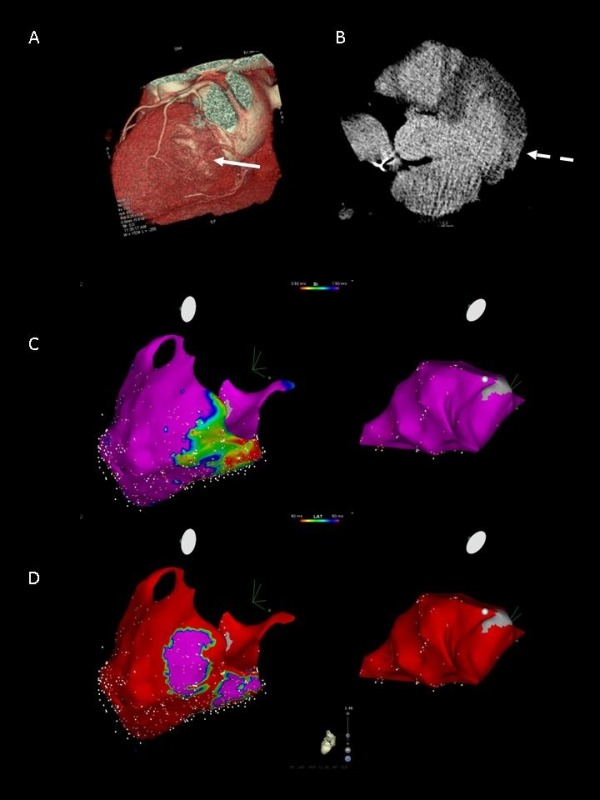
A case presenting good correlation between pathological area identified by cardiac CT and by electroanatomical map. A. Cardiac CT showed small scar in posterolateral wall in left ventricle B. Late enhancement of cardiac muscle was not seen in subendocardial region but seen in mesocardial and subepicardial portion. C and D. Electroanatomical mapping of epicardial space (left panel) and endocardial (right panel) of LV. The scar identified by CT was correspond to the border of low-voltage area in epicardial bipolar voltage map (C) and area with late potentials in local activation map (D) whereas there was no pathological region in endocardial map. (Radiofrequency energy was delivered epicardially. After abolishing all late potentials, VT was no longer inducible.)

The recent technological improvement of contrast enhanced CT has permitted detailed evaluation of the functional state and viability of ischemic and non-ischemic myocardium. Due to his high spatial ( <1mm) and temporal resolution some EP laboratories have begun to use this technology to improve VT ablation. A good correlation between areas of abnormal myocardium with low voltages (<1.5 mV) and CE-CT hypoperfusion was found . Interestingly CE-CT was also able to characterize the transmural extent and the intramyocardial location of the scar. Given this possibility, the better definition of the anatomical details and feasibility to perform this exam also in patients with an ICD it is possible that in the future CT-CT will be increasingly used in the setting of VT ablation (Figure 4).

In conclusion we think that, regardless of the type of technology used and the capability to best discriminate core scar from the border zone, the routine use of imaging before a VT ablation is mandatory to clearly identify the arrangement of the scars in order to plan the mapping and the ablation strategy.

## Conclusions

The epicardial ablation is an essential approach that must be available in an EP laboratory but the complexity of all the problems connected limits it extensive use only in high volume Centers provided of the necessary skillnes and competences. Only in this case this procedure can be considered applicable and may report a low rate of complications.

In the decision-making strategy the most important rule is carried out by the type of the cardiomyopathy. Patients with IDCM or ARVD should probably be submitted directly to an endo-epicardial approach while on the contrary ischemic patients should be submitted to an epicardial ablation only after a previous failed endocardial ablation.

In any case, a preprocedural evaluation with an imaging technique ( CT or MRI) is mandatory and must be considered as integral part of the decision-making process.

Informations obtained from the surface ECG, when available, are certainly useful but not pivotal.

Further research for development of dedicated energy delivery systems (to permit a selective and firmer contact with the epicardial layers and a reduction of possible collateral damage) and of new energy sources able to overcome the insulating properties of epicardial fat are desiderable.Further research for development of dedicated energy delivery systems (to permit a selective and firmer contact with the epicardial layers and a reduction of possible collateral damage) and of new energy sources able to overcome the insulating properties of epicardial fat are desiderable.

## Addendum

In the last 80 epicardial procedures we have routinely used the pressure monitoring obtained connecting a pressure line to the Tuohy needle.

**Figure 5:**
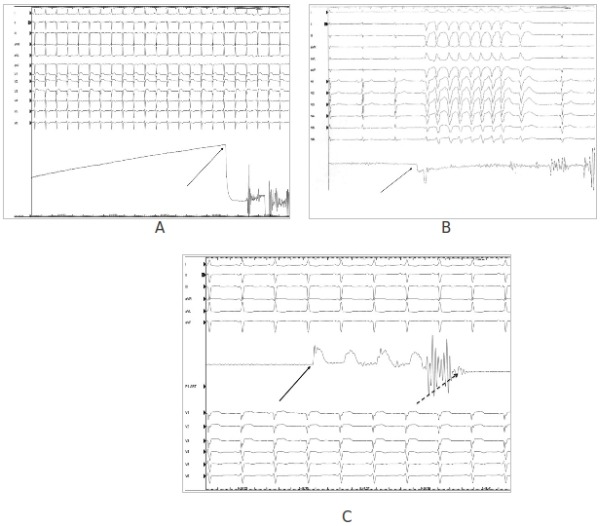
Use of the pressure monitoring during epicardial ablation is shown. A: Common pattern: initial progressive increase of the pressure during the movement of the needle toward the cardiac silhouette is followed by a sudden drop (arrow) when the tip of the needle get into the pericardial space. B: Unusual pattern: in some cases the increasing of the pressure is lower and also low is the drop of the pressure (arrow) when the needle reach the pericardial space. The contact of the needle tip with the myocardial wall usually induce VEB or, as in this case, VT. C: In some cases, particularly in the presence of soft adherence a drop of the pressure is not clearly visible and after the "click" the pressure of the right ventricle appears suddently (black arrow), suggesting that the neddle is in the right ventricle. In these cases, slight retraction of the neddle allows, when the right ventricle pressure curve disappears (broken arrow), the inserction of the wire in the pericardial space.

With this technique the needle must be advanced toward the cardiac silhouette with a slow and continuous movement. This induces a progressive increase of the registered pressure. Before entering the pericardial space a perception of the heart beat is always present and when the needle gets into the pericardial space (before that you frequently hear and feel a "click") a sudden drop of the pressure is clear on the pressure curve (Figure 5a). This drop is a distinctive sign that the tip of the needle is inside the pericardial space and is due to the negative pressure usually present in the pericardial space.

Sometimes the magnitude of the pressure drop is reduced (Figure 5b) (particularly in a non-empty pericardial space or in case of adherence) or you can feel two or three "clicks" with sudden change of the pressure, because you have to go across multiple layers (diaphragm, fibrous pericardium) before you reach to the pericardial space. In any case after a click and a pressure drop we check if the wire can procede in the pericardial space.

In some cases, probably due to very soft adherence, the "click" is immediately followed by the appearance of a pressure curve typical of the right ventricle (Figure 5c). The recognition of this condition prevents the insertion of the guide that in cases where the tip of the needle is still intramural could cause additional damage of the myocardial wall making the epicardial bleeding difficult to control in a non-surgical way. Moreover in these cases a very slow retraction of the needle until the RV pressure curve disappears allows in the vast majority of cases the advancement of the wire in the pericardial space. In this situation we start immediately with the epicardial mapping and anticoagulation for the endocardial mapping is started after half an hour. Bleeding in this condition usually stops in a few minutes and doesn't exceed 50 ml.
